# Early onset first episode psychosis: dimensional structure of symptoms, clinical subtypes and related neurodevelopmental markers

**DOI:** 10.1007/s00787-017-1026-7

**Published:** 2017-07-26

**Authors:** Maria Giuseppina Petruzzelli, Lucia Margari, Andrea Bosco, Francesco Craig, Roberto Palumbi, Francesco Margari

**Affiliations:** 1 Department of Basic Medical Sciences, Neuroscience and Sense Organ, Unit of Child and Adolescent Psychiatry, University of Bari “Aldo Moro”, Piazza Giulio Cesare 11, 70124 Bari, Italy; 2 Department of Educational Science, Psychology, Communication, University of Bari “Aldo Moro”, Piazza Giulio Cesare 11, 70124 Bari, Italy; 3 Department of Basic Medical Sciences, Neuroscience and Sense Organ, Unit of Psychiatry, University of Bari “Aldo Moro”, Piazza Giulio Cesare 11, 70124 Bari, Italy

**Keywords:** Schizophrenia, Early onset psychosis, Dimensions, Cluster analysis, PANSS, Neurodevelopment

## Abstract

Despite the growing interest in a dimensional approach to the assessment of symptoms and clinically relevant phenomena in schizophrenia spectrum disorders, very few studies, to date, have examined the dimensional structure of symptoms in early onset first episode psychosis. In the present study, we assessed a sample of 60 children and adolescents of both sexes with first episode schizophrenia spectrum psychosis. A principal component analysis (PCA) of the Positive and Negative Syndrome Scale (PANSS) was performed and the factors obtained were used to carry out a cluster analysis. Sex, age of onset before or after 13, markers of early neurodevelopmental impairment and intellectual disabilities were considered as variables to characterized potential clinical subtypes, applying a one-way analysis of variance. Four factors were extracted (“negative symptoms”, “delusions”, “conceptual disorganization” and “paranoid/hostility”), each of them identifying a discrete clinical subtype of patients. No difference was found among the groups about sex and age of onset; delayed speech/language development was significantly associated with the “delusions” subtype and both “conceptual disorganization” and “delusions” subtypes showed a lower intelligence quotient (IQ). The four factors model we presented highlights “negative symptoms” as the most consistent factor; among positive symptoms, unusual thought content and conceptual disorganization resulted more distinctive of psychosis, at this age range, than perceptual abnormalities. Evolutionary trajectories of the four clinical subtypes we obtained seem to be influenced by cognitive and neurodevelopmental impairment rather than sex and age of onset.

## Introduction

Early onset schizophrenia is a rare and severe condition in clinical and biological continuous with adult onset form of the disorder [1, 2]. The onset of schizophrenia in childhood is exceedingly rare (point prevalence <1/10.000 before the age of 12), but the incidence rises considerably in adolescence, and its prevalence is estimated at 0.23% in the age between 13 and 18 years [3, 4].

Studies exploring the effects of age of onset on clinical features in schizophrenia spectrum disorders showed that early and adult onset patients may differ in premorbid traits, illness presentation, clinical severity, and prognosis [3, 5–7]. Nevertheless, the diagnosis of schizophrenia spectrum disorders with onset in children and adolescents, up to date, is made using the same DSM-5 criteria than in adults, as reported by the American Academy of Child and Adolescent Psychiatry, AACAP [8]. The main changes from DSM-IV-TR to DSM-5 in the chapter “Schizophrenia Spectrum and Other Psychotic Disorders” introduced a conceptual psychosis continuum emphasizing the dimensions of psychosis [9, 10], but still defines psychotic disorder categories and does not make allowance for potential characteristics of patients in childhood and adolescence [11].

The dimensional model proposes that symptoms of psychosis tend to cluster together within different symptom complexes which can co-exist in individual patients [12]; thus, a dimensional assessment can allow for a more specific and individualized clinical evaluation of patients with schizophrenia spectrum disorders [9]. This can be very helpful at the onset of psychotic disorders, considering that the heterogeneity of clinical presentation can make more complicated treatment decisions and different aspects of research [13]. A major issue within the dimensional approach is the careful identification of the psychopathological dimensions, so that their specific etiological, physiopathological and clinical correlates may be identified [12]. Many authors have conducted dimensional studies using the Positive and Negative Syndrome Scale (PANSS) [14] in adult populations of patients with schizophrenia spectrum disorders [15–21]. The majority of dimensional studies agrees that 4 or 5 dimensions describe the psychosis construct, with positive, negative, disorganization and affective symptoms most frequently reported [22]. An important factor, which can influence the heterogeneity of results across studies, is the time frame of the study; a first episode psychosis (FEP) population may differ from a chronically ill population for symptoms profile and severity illness, so as the dimensional structure of FEP may be affected by early or adult age of onset [17, 22].

To our knowledge, very few studies have examined the dimensional structure of symptoms in childhood and adolescence psychosis, using different rating scales (Scale for Assessment of Positive Symptoms, Scale for Assessment of Negative Symptoms, Brief Psychiatric Rating Scale, Schedule for Affective Disorders and Schizophrenia-for School Age Children, PANSS) and includung patients with differnt diagnosis at baseline (schizophrenia and related disorders, mood disorders, anxiety disorders) [23–26]. Only one among these examined the dimensional structure of symptoms in early onset psychosis through a principal component analysis (PCA) of the PANSS [26].

In the present paper, we studied a group of patients with FEP below 18 years, applying a three-step dimensional approach:

1. To examine the dimensional structure of symptoms through a PCA of the PANSS;
2. To explore whether specific clinical subgroups of patients can be identified on psychopathological basis by a cluster analysis;
3. To search for correlations between such identified subgroups of patients and some clinical markers having a potential influence on illness presentation, as sex, age of onset before or after 13, markers of neurodevelopmental impairment and intellectual disabilities.

## Materials and methods

### Subjects

The study sample included 60 patients of both sexes, less than 18 years of age, consecutively referred over a 6-year period, since 2008, among the inpatients of the Child Neuropsychiatry Unit, Department of Basic Medical Sciences, Neurosciences and Sense Organs, University of Bari “Aldo Moro”, Italy. They were antipsychotic-naïve patients in their FEP, defined as the manifestation of delusion, hallucination and/or disorganization symptoms of less than 6 months’ duration at the time of the assessment. Diagnoses of early onset first episode schizophrenia spectrum psychosis (schizophrenia, schizophreniphorm disorder, schizoaffective disorder, psychosis not otherwise specified) were made in accordance with DSM IV-TR criteria, and supported by using the italian version of the semi-structured diagnostic interview, Kiddie-Schedule for Affective Disorders and Schizophrenia-Present and Lifetime Version, (K-SADS-PL); experienced psychiatrist trained in the use of instrument interviewed parents and patients. Premorbid and actual functioning was assessed using, respectively, the Premorbid Adjustment Scale (PAS) [27] and the Children’s Global Assessment Scale (CGAS) [28]. Data on early signs and symptoms of psychosis were collected to define the temporal order and pattern of premorbid course. To this purpose, we carried out a retrospective interview with patients and their relatives, focusing on the onset of prodromal symptoms of psychosis, cognitive and social impairments; information from the past and present clinical reports was also noted. Patients were also subjected to a comprehensive clinical assessment, including an anamnestic interview, a physical and neurological examination, as well as an instrumental evaluation by laboratory blood tests, electrocardiogram, electroencephalogram and brain magnetic resonance. Exclusion criteria consisted of history of traumatic brain injury with loss of consciousness, cerebral tumours and evidences from the history, physical examination or laboratory findings that the psychotic disorder was substance induced or due to another medical condition. After providing complete description about the study, we obtained written informed consent from the parents of all subjects. Ethical Committee of the Hospital Consortium Policlinic of Bari, Italy, approved the study (prot. n. 0075163).

### Assessment of psychopathology

The psychopathological evaluation was performed by the validated italian version of the PANSS [29], a standardized instrument including 30 items on a seven-point scale to assess positive (7 items), negative (7 items) and general symptoms (16 items). The PANSS is widely used in clinical and research settings and is regarded as a reliable means of a comprehensive assessment of the symptoms of schizophrenia [16]. All patients were clinically assessed within the first 72 h after their admission to child neuropsychiatry unit, by two members of research staff, receiving training in the use of the PANSS. The assessment was performed following a semi-structured interview to ensure that all content domains were covered during the evaluation session; the scores were assigned according to PANSS rating criteria, and all the evaluations were discussed in regular reliability meetings, supervised by the senior researcher.

### Early neurodevelopmental impairment

At least one of the parents was interviewed to record early childhood neurodevelopmental milestones: motor, speech, sphincter control, social skills and school learning. An impairment in each of these areas of development was scored as present or absent using a modified version of General Developmental Scale, GDS, previously published in more detail [30, 31].

### IQ measures

All subjects underwent an intelligence testing for the measurement of IQ. The first choice was the Wechsler Intelligence Scale for Children-Revised (WISC-R) [32], which provides a measure of verbal, performance and full-scale IQ. For the patients with speech and language difficulties, according to the clinical judgment, a nonverbal test was used, the Leiter International Performance Scale-Revised (Leiter-R), battery of visualization and reasoning [33]. Both scales allow distinguishing patients with normal IQ from patients with borderline IQ, mild, medium and severe cognitive impairment.

### Statistical analysis

A PCA was carried out with the principal aim to assemble items of PANSS in a series of coherent components able to tackle psychopathological dimensions in patients with early onset psychosis. The best factorial solution should be able to keep adequate statistical parameters and theoretical/interpretative aspects, as well. All thirty PANSS items (7 Positive, 7 Negative and 16 General) were taken into account and four components were extracted. Items with an unclear factor loading distribution (i.e., they showed similar factor loadings in more than one component) or with low factor loadings (<0.45) were excluded by the components. The final component solution was first obliquely rotated (OBLIMIN method) to exclude the likelihood of highly correlated components and, subsequently, orthogonally rotated (VARIMAX method) increasing the statistical interpretability of the final item arrangement. Afterwards standardized component scores were used (a) to explore, within the sample, the presence of subgroups of patients with similar psychopathological profile performing a hierarchical cluster analysis (Ward’s method). This model of analysis computes sum of squared distances between scores of patients within each cluster and iteratively aggregate clusters with the minimum increase in the overall distance across the variables considered, and (b) a k-means cluster analysis (initial cluster center method: choose observations to maximize initial between-cluster distances). This procedure has the purpose of minimizing the within-cluster and maximizing the between-cluster variability, so that the differences between means for each examined variable are the highest between clusters. It is worthwhile to note that the examined variables were the standardized component scores of the former PCA. The hierarchical cluster analysis was used with an exploratory while the k-means with a confirmatory aim. Finally, in order to examine univariate associations between clusters and other outcome variables, a series of one-way ANOVAs were performed for discrete numerical dependent variables. Chi Square was adopted for nominal variables (i.e., gender and age of the first episode dichotomized in until to versus more than 13-years-old).

## Results

### Sample features

Demographic and clinical features of the sample are resumed in Table 1. Only 8.3% of patients had an acute onset of psychotic symptoms, while the remaining 91.7% presented a slower and gradual impairment of psychopathology, up to full psychotic symptoms. The mean duration of the illness, meant as the difference from time of onset of psychotic symptoms to the PANSS evaluation, was 3.2 months (±1.8 SD). At the time of the enrollment, 28.4% of the sample were already receiving psychopharmacological treatment other than antipsychotics: benzodiazepines (16.7%), antidepressants (6.7%), mood stabilizers (5%). PANSS total score was 95 (±8.19 SD), while PANSS subscale scores were 16 (±8.26 SD) for positive symptoms, 22 (±2.84 SD) for negative symptoms, 50 (±5.95 SD) for general psychopathological symptoms. Data on the early childhood neurodevelopmental milestones showed delayed motor development in 12.7% of patients, delayed language development in 25%, delayed sphincter control in 24%, impaired social development in 46%, school learning difficulties in 44%. Five patients were unable to complete the IQ testing because of their clinical conditions. We found an intellectual functioning level below average in 52.72% of the sample (27.59% borderline, 37.93% mild, 27.59% medium and 6.89% severe cognitive impairment).

**Table 1:** Demographic and clinical features of the sample

*N* = 60	
Sex, *n* (%)	
Male	37 (61.7)
Female	23 (38.3)
Age, mean (SD)	14 (2.88)
Age < 13 *n* (%)	16 (26.7)
Age 13–18 *n* (%)	44 (73. 3)
Obstetric complications, *n* (%)	26 (43.3)
Family history of psychiatric disorders^a^, *n* (%)	38 (63.3)
PAS mean (SD)	0.55 (0.15)
C-GAS mean (SD)	39 (7.42)
IQ mean (SD)	78.87 (23.15)
Diagnosis *n* (%)	
Schizophrenia	18 (30)
Schizophreniphorm disorder	8 (13.33)
Schizoaffective disorder	10 (16.67)
Psychosis not otherwise specified	24 (40)

^a^ Schizophrenia and related disorders, mood disorders, anxiety disorders, substances abuse/dependence, personality disorders, and mental retardation

### Psychopathological dimensions

PCA was applied to the PANSS scale (KMO value = 0.73, Bartlett’s Test of Sphericity *p* < 0.01); four components were extracted explaining 47% of the total variance (Table 2). The four components had eigenvalues ranging from 2.81 to 4.44 after a VARIMAX rotation. Preliminarily, an OBLIMIN rotation was also performed. All correlations between components were under 0.3. In light of this result, the components were considered uncorrelated, then a VARIMAX rotation was performed. The four components had eigenvalues ranging from 2.81 to 4.44. They showed to be adequately reliable since Cronbach’s Alphas ranged from 0.72 (fourth component) to 0.84 (first component). The first factor was named “negative symptoms” and was composed by the following items: blunted affect (N1), emotional withdrawal (N2), lack of spontaneity and flow conversation (N6), active social avoidance (G16), poor rapport (N3), passive/apathetic social withdrawal (N4). The second factor was named “delusions” and was composed by the following items: unusual thought content (G9), stereotyped thinking (N7), preoccupation (G15), delusions (P1), lack of judgment and insight (G12). The third factor was named “conceptual disorganization” and was composed by the following items: anxiety (G2), tension (G4), difficulty in abstract thinking (N5), motor retardation (G7), somatic concern (G1), conceptual disorganization (P2). The fourth factor was named “paranoid/hostility” and was composed by the following items: uncooperativeness (G8), hostility (P7), suspiciousness/persecution (P6), poor impulse control (G14), excitement (P4). According to the regression method (see “Statistical analysis” section), four standardized component scores were calculated, one for each component.

**Table 2:** Results of principal components analysis of PANSS items showing factor loadings, eigenvalues, percentage of explained variance

	Factor 1	Factor 2	Factor 3	Factor 4
N1 blunted affect	**0.786**	0.12	0.116	0.156
N2 emotional withdrawal	**0.766**	−0.019	0.055	0.215
G16 social avoidance	**0.709**	−0.153	−0.08	−0.02
N6 lack of spontaneity	**0.657**	−0.001	0.013	0.222
N3 poor rapport	**0.648**	−0.347	−0.019	−0.346
N4 social withdrawal	**0.624**	−0.405	0.012	−0.292
P5 grandiosity	−0.557	0.098	0.242	0.205
G6 depression	0.446	0.243	−0.063	0.058
G13 disturbance of volition	0.34	0.169	0.313	0.224
G9 unusual thought content	−0.273	**0.742**	0.04	0.066
N7 stereotyped thinking	0.051	**0.682**	−0.178	0.022
G15 preoccupation	0.289	**0.632**	−0.486	−0.029
P1 delusion	0.077	**0.604**	−0.014	0.093
G12 lack of insight	−0.07	**0.559**	0.126	0.169
G5 mannerisms and posturing	0.033	0.379	0.264	−0.021
G10 disorientation	−0.174	0.358	0.325	0.082
G2 anxiety	0.107	−0.181	**0.746**	0.03
G4 tension	0.129	−0.189	**0.745**	−0.02
N5 difficulty in abstract thinking	−0.128	0.137	**0.619**	−0.158
G7 Motor retardation	0.339	0.143	**0.584**	−0.072
G1 somatic concern	0.159	−0.552	**0.581**	0.087
P2 conceptual disorganization	−0.259	0.217	**0.484**	−0.09
P3 hallucinatory behavior	−0.102	−0.007	0.31	−0.007
G8 uncooperativeness	0.123	0.19	−0.113	**0.795**
P7 hostility	0.162	0.001	−0.062	**0.78**
P6 suspiciousness	0.108	0.396	−0.025	**0.584**
G14 poor impulse control	−0.349	−0.185	0.049	**0.529**
P4 excitement	−0.417	−0.03	0.414	**0.456**
G3 Guilt feelings	0.196	−0.137	−0.243	0.396
G11 poor attention	0.028	−0.093	−0.019	−0.258
Eigenvalue	4.44	3.5	3.4	2.81
Percentage of variance	14.79	11.66	11.34	9.36

Bold font identifies the component to which the item is attributed

### Psychotic clusters

Using the four standardized component scores mentioned above, we performed a preliminary cluster analysis (Ward’s Method), following by a k-means cluster analysis to confirm the model. They converged highly on a solution with four subgroups based on different patterns of symptoms. Each psychopathological dimension led to the identification of a specific subgroup of patients (Table 3): Cluster 1, composed by 25 patients with the highest score in conceptual disorganization, Cluster 2, composed by 12 patients with the highest score in delusions and very low score in negative dimension, Cluster 3, composed by 13 patients with the highest score in negative symptoms, Cluster 4, composed by 10 patients with the highest score in paranoid/hostility. Table 3 summarizes dimensional contributions to four psychotic subtypes emerged from the cluster analysis.

**Table 3:** PANSS clusters

Factors	Cluster 1 (*n* = 25)	Cluster 2 (*n* = 12)	Cluster 3 (*n* = 13)	Cluster 4 (*n* = 10)	*F* (3; 56)	*p*	Eta_*p*_^2^
	M (SD)	M (SD)	M (SD)	M (SD)			
Negative symptoms	−0.26 (0.65)	−0.92 (0.55)	**1.18 (0.95)**	0.23 (0.70)	19.94	<0.001	0.52
Delusions	−0.46 (0.64)	**1.06 (0.85)**	−0.12 (1.07)	0.03 (0.96)	8.94	<0.001	0.32
Conceptual Disorganization	**0.57 (0.90)**	−0.20 (0.83)	−0.66 (1.10)	−0.31 (0.48)	6.43	<0.001	0.26
Paranoid/hostility	−0.23 (0.60)	−0.34 (0.89)	−0.56 (0.56)	**1.70 (0.51)**	28.51	<0.001	0.60

Characteristic symptoms

*N*, Means ± standard deviations of the standardized component scores obtained by the PANSS Clusters. *F* ratio (*df*), *p* and partial eta square values were also reported. The score characterizing the cluster is in bold. Standardized component scores were calculated on the basis of the Regression Method (e.g., Mulaik 2009)

### Univariate associations with psychotic clusters

The distribution of males and females across the four subgroups of patients was not significantly different (Chi square = 2.95; *p* > 0.1), as well as age of onset of the first episode psychosis, dichotomized as until to/after 13 years (Chi square = 3.57; *p* > 0.1).

Among the five areas of early childhood neurodevelopmental, we found delayed speech/language development significantly higher in “delusions” subtype; moreover, Fisher LSD post hoc tests showed that this characteristic is important to discriminate “delusions” by “negative symptoms” and “paranoid/hostility” subtypes (Table 4).

**Table 4:** Relationship between PANSS clusters and neurodevelopmental markers

Markers of neurodevelopmental impairments	Cluster 1 (*n* = 25) conceptual disorganization	Cluster 2 (*n* = 12) delusions	Cluster3 (*n* = 13) negative symptoms	Cluster 4 (*n* = 10) Paranoid/ hostility	F(3,56)	*p*	*η*_*p*_^2^	Post hoc tests^a^
Impaired social development	−0.03 ± 0.68	0.17 ± 0.7	−0.1 ± 0.68	0.19 ± 0.7	0.55	0.65	0.03	
Delayed motor development	−0.05 ± 0.35	0.46 ± 0.87	0.01 ± 0.49	0.05 ± 0.56	2.51	0.07	0.12	
Delayed language development	0.12 ± 0.67	0.51 ± 0.8	−0.14 ± 0.43	−0.25 ± 0	3.74	0.02	0.17	Cluster 2 > 3 = 4
Delayed sphincter control	0.12 ± 0.67	0 ± 0.6	−0.02 ± 0.58	0.21 ± 0.74	0.31	0.82	0.02	
School learning difficulties	0.16 ± 0.69	0.19 ± 0.7	−0.08 ± 0.69	−0.33 ± 0.57	1.6	0.2	0.08	
Cognitive impairment	0.19 ± 0.62	0.12 ± 0.67	−0.05 ± 0.7	−0.51 ± 0.57	3.05	0.04	0.14	Cluster 1 = 2 > 4

Means ± standard deviations of scores tackling neurodevelopmental markers for each cluster of patients. Mean values are to be intended as *z* scores (*z* = [*x*−*m*]/sd). A one-way Anova for each marker as dependent variable is performed. *F*, *p* and effect size (partial eta square) values are reported as well

^a^ Fisher LSD tests are performed. Lacking comparisons have to be intended as not significant

A significantly lower IQ was found in both “conceptual disorganization” and “delusions” subtypes than in “paranoid/hostility” subjects (Table 4).

## Discussion

The main finding of this study was a four-factor model describing latent psychopathological dimensions resulting by the use of the PANSS in a sample of FEP patients with onset before 18. This solution can be considered an adequate model since it accounts for 47.15% of the total variance and the internal consistency for each factor is good. The four factors were “negative symptoms”, “delusions”, “conceptual disorganization” and “paranoid/hostility”, and each of them proved to identify a discrete clinical subgroup of patients by a cluster analysis. Furthermore, we found that delayed speech/language development was significantly associated with the “delusions” subtype, and that “conceptual disorganization” and “delusions” subtypes showed both a lower IQ than the other subtypes.

Factor analytic solutions of rating scale are in part scale dependent, disease dependent and state dependent; thus, variables as age of onset, diagnoses, acute or chronic phase of disease could modify the results between different studies [34]. Rapado-Castro et al. examined the dimensional structure of symptoms in early onset psychosis applying a PCA of the PANSS; they found a five-factor solution (positive, negative, depression, cognitive, hostility) suggesting a continuum between early and adult-onset form of the disorders [26]. Examining the four factors solution emerged from our study, we have found a substantial overlap with the Rapado-Castro model. “Negative symptoms” were the most consistent factor we found, accounting for the highest percentage (14.79%) of the total variance, as also reported by Rapado-Castro et al. Moreover, the negative dimension was found to be the most robust even in those studies exploring the dimensional structure of symptoms by the PANSS in samples of patients with adult onset first episode psychosis [16, 17, 35]. The other three components we found were each focused on a different aspect of the positive symptomatology. The factor named “delusions” was characterized by unusual thought content, stereotyped thinking, preoccupation, delusions, lack of judgment and insight. The “conceptual disorganization” dimension was focused on formal thought disorders, associated with a physical and psychical somatic component, including difficulty in abstract thinking, conceptual disorganization, motor retardation, anxiety, tension, somatic concern. The “paranoid/hostility” factor combined behavioral symptoms with suspiciousness and persecution, namely uncooperativeness, hostility, suspiciousness/persecution, poor impulse control, excitement. We noted that the “hallucinatory behavior” item has not significantly contributed to any of the four components, having a factor load of 0.31, lower than the indicated value as cutoff. “Hallucinatory behavior” also had a low factor load (0.41) in Rapado-Castro study, whereas higher scores, between 0.65 and 0.806, have been found in studies on adult onset FEP [16, 17]. As introduced by DSM 5, the primary symptoms of psychosis are on a continuum, so we can define a gradient of severity between different patients and, over the time, in the same subject [9]. Moreover, populations-based epidemiologic studies have shown that auditory hallucinations are common particularly in young age groups with prevalence rates ranging from 5.7 to 21.0%. For the majority of children and adolescents, these experiences are well below the threshold of clinical disorders and tend to have a benign course [36–38]. On the other hand, no other clinical phenomena are as likely to be attributed to schizophrenia as hallucinations, yet no other clinical phenomenon common to schizophrenia is as widely spread across numerous other states and disorders, including attention deficit-hyperactivity disorder, conduct disorders, depressive and anxiety disorders, post-traumatic stress disorder, obsessive–compulsive disorder, and borderline personality disorders [36]. It is also notable that hallucinations and delusions, traditionally considered to be evidence of impaired reality testing and gathered together in a single dimension, have been designed better as separate dimensions by DSM-5 [39]. This distinction could be particularly useful in adolescent patients, as suggested by our data in which formal and content thought disorders appeared to be independent of the hallucinatory symptoms. To further support this assumption, Lehembre-Shiah et al. found that only unusual thought content and thought disorganization, not perceptual abnormalities, are associated with conversion to psychosis in clinical high-risk populations, despite the high incidence of perceptual abnormalities [40]. We can argue that, especially at this age range, hallucinations may be considered as transdiagnostic symptoms; thus, the clinical relevance of perceptual abnormalities must take into account markers of severity, comorbid psychopathology and levels of functioning of young patients. This dimensional approach should have a central issue in clinical practice to improve decision making on psychopharmacological and psychosocial interventions in early phase of psychosis. Lastly, compared to the Rapado-Castro model, the solution we obtained does not contain the depressive dimension. This difference may be caused by selection of the sample, since we enrolled patients with non-affective psychosis, while 24.2% of the Rapado-Castro study sample included patients with mood disorders (bipolar and depression) as diagnosis at baseline.

When we used the four factors describe above as a summary of symptoms in cluster analysis, we found that each of these four psychopathological dimensions can identify a discrete clinical subtypes of patients with FEP. This result appears in line with the direction of the DSM-5 that suggests using psychopathological dimension to improve the ability to describe the clinical heterogeneity of schizophrenia, overcoming the traditional DSM-IV subtypes (paranoid, disorganized, catatonic and undifferentiated) [41]. Growing evidences are showing as a dimensional approach to symptoms domains in schizophrenia is more valid and clinically useful in describing clinical picture; in addition a dimensional approach may provide more relevant informations to lead diagnosis and treatment decision [42–44].

The third aim of this study was to characterize the four clinical subtypes of early onset FEP emerging from the cluster analysis, through the association with sex, age of onset, markers of early neurodevelopment abnormalities and intellectual disability. We found differences between the four groups regarding the early speech and language development and the cognitive profile. Consistent with the neurodevelopmental hypothesis of schizophrenia, premorbid dysfunctions (including language, motor and social deficits) have been well documented in patients with the disorder and were found to be more frequent in subjects that will later on develop schizophrenia during childhood or adolescence, compared to adulthood [4, 45]. The effort of actual and prospective studies is to improve understanding about how psychobiological markers change over time in young people at varying degrees of risk for psychosis and link them to differential clinical, functional or other end points [46]. We found that “delusions” subtype patients had more significant delayed language development than patients in other clinical subtypes. Language dysfunction plays a central role in the clinical presentation of psychosis, but there is a gap in our understanding of the longitudinal development of speech from childhood and of its relationship with the evolution of at risk mental state and psychotic illness [47]. Children with developmental speech/language impairments are at high risk for literacy skills [48]; Hameed et al. found that low performance in literacy skills (spelling, basic real and non-real word reading, reading skills and comprehension), assessed between 7 and 9 age, and a declining pattern over time, was associated with psychotic experiences in early adolescence [49]. Based on our data and of these previous literature findings, we can hypothesize an evolutionary continuity between early alterations of language development, impairment of literacy skills and delusional dimension. Furthermore, a large amount of data suggest that cognitive deficits can be considered as vulnerability markers of psychosis and a core feature of schizophrenia [50, 51]. Some authors suggest that cognitive profiles are quite heterogeneous in patients at FEP indicating quantitative and qualitative differences across the schizophrenia spectrum disorders subgroups [52]. We found that adolescents with cognitive impairment are more likely to develop delusions and conceptual disorganization rather than negative symptoms or paranoid/hostility. This finding could suggest either that the cognitive profile can influence the clinical presentation of psychosis, or that a specific relationship exists between the cognitive impairment and some psychopathological dimensions of psychosis. There is, indeed, considerable evidence for reasoning, attention, metacognition and attribution biases in people with delusions or prone to delusional thinking. The strongest evidence base relates to the “jumping to conclusion” and attributional biases. Recently, these findings have been incorporated into a number of cognitive models that aim to explain delusion formation, maintenance and content within a model of impairment of normal belief formation [53–55].

Some methodological limitations should be recognized. First of all, regarding the factor analysis we have to consider that the results may be influenced by the measurement instrument and that, according to the items composition of the factors we obtained, slight differences in nomenclature with respect to other studies are been used. In addition, the relatively small sample size would have minimize statistical power and limited our ability to detect subgroup differences. However, the sample was relatively homogeneous for diagnosis, the extracted components showed an adequate internal consistency and the factor loadings of all components were pretty high (>0.5 except for two of them); therefore, the proposed solution can be considered reasonable. Since, at present, contributions on the topic of psychopathological dimension of early onset psychosis are rare, studies on relatively small samples can be worthwhile in contributing to the comprehension of the disorder, although the data are not generalizable. Second, we have to report the lack of inter-rater reliability analyses for the PANSS assessment. Third, although the sample study is antipsychotic-naive, some patients had been exposed to small quantities of benzodiazepines, antidepressants and mood stabilizers, so that the sample cannot be regarded as totally medication-naive.

In conclusion, we presented a four-factor solution describing psychopathological dimensions in childhood and adolescent FEP. “Negative symptoms” was the most robust component; the other three dimensions were “delusions”, conceptual disorganization” and “paranoid/hostility”; hallucinator behavior was not included in any of components. These data could suggest that, among positive symptoms, unusual thought content and thought disorganization may be more distinctive of early onset psychosis than perceptual abnormalities. Moreover, we found that the psychopathological dimensions identified four corresponding clinical subgroups in the study sample, confirming that the dimensional approach may offer a better conceptualization of FEP also in children and adolescent patients. Further replication across additional sample would be needed to confirm this factor solution, in order to generalize the dimensional model to all subjects with early onset schizophrenia spectrum disorders. Finally, this study showed a relationship between the different clinical subtypes and cognitive impairment and speech/language development abnormalities. Longitudinal research designs should be stimulated to capture changing aspects of clinical and neurobiological markers, starting from early stages of neurodevelopment until the onset of overt psychotic symptoms. This evolutive approach would be very useful in early intervention programs to guide risk assessments and treatment decisions of children and adolescents at risk of psychosis.
